# A Case Study Based on the Effect of Phytoestrogen on an Oligozoospermic Patient

**DOI:** 10.7759/cureus.54071

**Published:** 2024-02-12

**Authors:** Ritesh Jadhav, Akash More, Namrata Choudhary, Jarul Shrivastava, Shilpa Dutta, Gauri Gajabe, Saurabh Mehakar

**Affiliations:** 1 Clinical Embryology, School of Allied Health Sciences, Datta Meghe Institute of Higher Education and Research, Wardha, IND; 2 Clinical Embryology, Wardha Test Tube Baby Centre, Datta Meghe Institute of Higher Education and Research, Wardha, IND

**Keywords:** varicocele, intrauterine insemination, infertility, semen, phytoestrogen, severe oligospermia

## Abstract

We presented a 30-year-old man suffering from severe oligozoospermia caused by substantial sperm maturation arrest around the spermatid stage. Additionally, he was suffering from a varicocele. For three years, the couple had been trying to conceive. The clinical and endocrinological evaluation of the woman revealed that she was medically fit to undergo pregnancy. We performed laparoscopic ligation of the spermatic vein to treat the varicocele. Semen analysis was conducted at the beginning of the clinical pregnancy journey and after three and six months of treatment, which included 80 mg/day of phytoestrogens for six months. Six months following the end of the therapy, a second semen analysis was carried out. The inherent characteristics of the semen substantially improved in the third month, facilitating the implementation of the reproductive method referred to as intrauterine insemination. Following this treatment, the patient delivered a healthy baby weighing 3300 g.

Sperm parameters improved substantially after three months of therapy; however, they reverted to baseline values during the wash-out period. These promising findings strongly suggest that phytoestrogens could be utilized for therapeutic purposes in the management of oligozoospermia. To further demonstrate the potential impact of phytoestrogens on male infertility, it is imperative to conduct a validation phase and randomized controlled trials.

## Introduction

Male infertility frequently manifests with significant oligozoospermia as a primary contributor. A comprehensive assessment involves a systematic and thorough investigation of potential underlying causes, such as hormonal imbalances, genetic factors, anatomical abnormalities, infections, lifestyle factors, and environmental exposures. This facilitates targeted management strategies for optimal outcomes [1]. The most crucial aspect is pinpointing the precise cause and determining potential interventions. Seminal plasma examination is the cornerstone diagnostic tool for this condition, guiding various biological tests, including serum hormone level measurements [2]. The male genital tract secretes different chemicals crucial for reproductive function. These include zinc from the prostate, transferrin from Sertoli cells, carnitine signalling epididymal activity, and fructose from seminal vesicles. Each substance serves a distinct role, collectively contributing to the composition and functionality of seminal fluid. In the context of varicocele treatment, it's essential to consider its potential impact on the secretion and composition of these crucial chemicals within the male genital tract. Varicocele can disrupt the microenvironment of the testes, potentially affecting the secretion and balance of substances like zinc, transferrin, carnitine, and fructose. Addressing varicocele through interventions such as ligation of the spermatic vein can help restore the physiological environment within the male reproductive system, thereby potentially optimizing the secretion and functionality of these substances [3]. Our research endeavours to categorize oligozoospermia types and evaluate associated biochemical factors, thereby assessing the extent of dysfunction. By meticulously analysing these parameters, we aim to gain insights into the underlying mechanisms and potentially inform therapeutic interventions for individuals experiencing fertility challenges [4]. Severe oligozoospermia, defined by a sperm count of less than five million, poses significant challenges to fertility in men. Various factors, including genetic anomalies, hormonal imbalances, and lifestyle habits like smoking, alcohol consumption, and exposure to environmental pollutants, may contribute to its development [4]. Treatment strategies for severe oligozoospermia revolve around pinpointing and rectifying root causes. Medications might be recommended to address genetic or hormonal abnormalities. At the same time, lifestyle adjustments, like quitting tobacco, limiting alcohol consumption, and reducing exposure to environmental toxins, can play a pivotal role in enhancing sperm count. Additionally, dietary changes and stress management techniques may be implemented to optimize reproductive health. By addressing both medical and lifestyle factors, individuals with severe oligozoospermia can potentially enhance their chances of achieving successful conception [5]. This case report holds significance as it sheds light on novel methodologies for improving sperm count in oligozoospermia patients.

## Case presentation

Patient information

We present the case of a 30-year-old South Asian male patient who visited our centre, the Wardha Test Tube Baby Centre, Wardha, India, in August 2020, showing infertility and a left-sided varicocele. He had no history of lifestyle disorders, had abstained from alcohol, was non-diabetic, and had no history of smoking or surgeries. There was no significant family history of medical conditions.

Clinical findings

During the physical examination, the patient's varicocele was classified as grade 3 according to a three-grade classification system [5] (grade 1: only detectable during the Valsalva manoeuvre; grade 2: perceptible at rest; grade 3: visible at rest). The reflux was determined to be severe when the patient was supine at rest. Reflux was categorized during colour Doppler ultrasound into three types: mild, transient reflux terminating before completion of the Valsalva manoeuvre, persistent reflux during the Valsalva manoeuvre, and severe reflux.

There was no history of smoking, drug use, or systemic diseases. Hormonal markers remained within normal ranges both pre and post surgery. The couple had attempted to conceive for three years without success. Semen analysis revealed severe oligozoospermia, consistent with the World Health Organization guidelines, with a median proportion of progressive motile sperm of 48% and a median percentage of standard forms of 32%.

**Figure 1:**
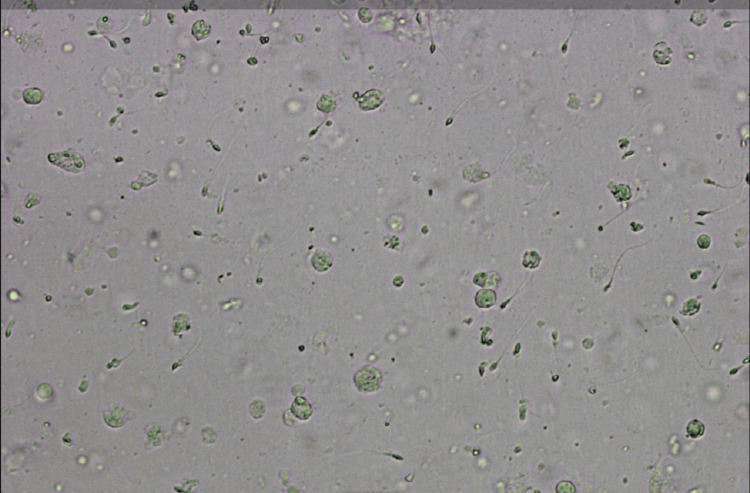
Image of semen sample showing severe oligozoospermia (2 million/mL) and few pus cells Image Credit: Author

The analysis of the patient revealed severe oligozoospermia, with a count of 2 million/mL, as shown in Figure 1. Furthermore, the patient's wife exhibited a healthy history, a normal hormone status, and a hysterosalpingogram indicative of normal findings.

Treatment

The male patient with infertility and left-sided varicocele underwent laparoscopic ligation of the spermatic veins. Grade 3 varicocele with severe reflux was noted. Post surgery, phytoestrogen therapy (80 mg/day for six months) was initiated. Follow-up at one, three, and six months showed improved semen parameters. Intrauterine insemination (IUI) was performed, resulting in successful clinical pregnancy. This case demonstrates the efficacy of laparoscopic intervention and postoperative phytoestrogen therapy in addressing male infertility. After treatment with phytoestrogen therapy, semen analyses were conducted three times each month.

Follow-up

**Figure 2:**
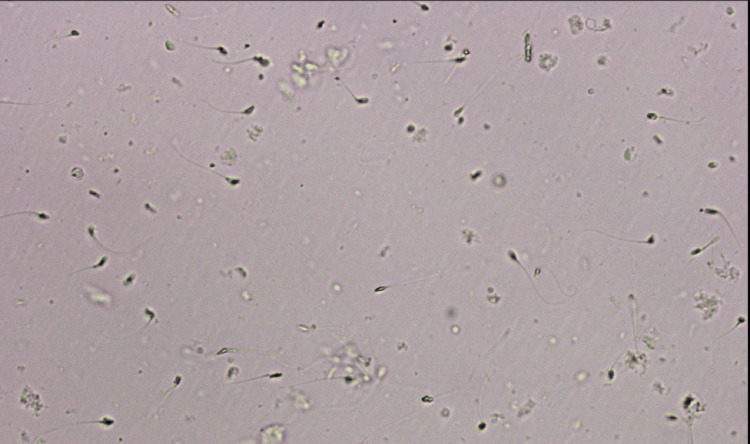
Semen sample after treatment with phytoestrogen. Sperm count is 8 million/mL Image Credit: Author

A follow-up physical examination was conducted on the patient one-month post surgery, accompanied by spermatic colour Doppler and semen analysis at the third and sixth months, revealing improved semen parameters, as shown in Table 1 and Figure 2. Before phytoestrogen therapy, semen analysis indicated a low sperm count of 2 million/mL, a 48% motility, and a high defect rate of 92%, with only 8% normal morphology. Following the intervention, there was a significant increase in sperm count to 8 million/mL, alongside improved motility (60%) and morphology (15%), coupled with a reduced defect rate to 85%.

**Table 1:** Quality of male reproductive cells before and after intervention

Semen analysis before intervention	
Semen parameter	Value after analysis
Sperm count	2 million/mL
Motility	48%
Defect	92%
Normal morphology	8%
Semen analysis after intervention	
Sperm count	8 million/mL
Motility	60%
Defect	85%
Normal morphology	15%

Subsequently, IUI was performed using the processed semen sample, resulting in a favorable clinical pregnancy outcome for the couple.

This scientific description details the laparoscopic procedure for spermatic vein ligation, the postoperative management involving phytoestrogen therapy, and the ensuing reproductive outcomes following IUI. Each line seamlessly connects to the next, providing a coherent narrative of the clinical interventions and their results.

## Discussion

Severe oligozoospermia, characterized by an abnormally low sperm concentration in semen, poses significant challenges to male fertility and reproductive health. Diagnosis typically involves a semen analysis revealing a sperm count of less than 5 million/mL of semen. This condition can make conception difficult for couples trying to conceive. In addition to the reduced sperm count, individuals with severe oligozoospermia may experience a range of symptoms and associated factors. These can include decreased libido, erectile dysfunction, testicular pain or swelling, and hormonal imbalances such as low testosterone levels. Seeking guidance from healthcare professionals specializing in reproductive health is crucial for those facing fertility challenges due to severe oligozoospermia.

When used to treat severe oligospermia, phytoestrogens were successful in terms of both improving seminal parameters and the success of the couple getting pregnant after the initial IUI. Additionally, semen parameters were restored to baseline levels after six months of wash-out, demonstrating that there is an association with phytoestrogen therapy [1]. Currently, the impact of oestrogens on spermatozoa development has been thoroughly studied [6]. We know that the testis may generate oestrogens and react to them at all stages of development [7]. The distribution of endoplasmic reticulum (ER) subtypes and aromatases in the testis shows that oestrogen is crucial in testicular and epididymal function [5]. Regarding sperm capacitation and acrosome responses, genistein and flavanone phytoestrogen 8-prenylnaringenin improved sperm fertility in in vitro submicromolar quantities of mouse sperm [5]. The potential of environmental oestrogens, such as genistein, to dramatically enhance mammalian sperm capacitation and fertilising ability is greater than that of oestrogen (E2) [8]. To explain how phytoestrogens alter some processes that may have an impact on male fertility, an ER-independent mechanism has also been proposed [9]. In their research, Jafari and colleagues assessed the effects of genistein and daidzein on cultured adrenal cortical cells. It would be reasonable to infer that the modulation of steroidogenic activity (lower cortisol production and enhanced androgen synthesis) appears modulated by extra-genomic pathways, presumably by direct modulation of steroidogenic enzyme activity [10].

To address hormonal imbalances or physical abnormalities, including varicoceles (enlarged veins in the scrotum), hormone therapy or surgery may be recommended. A baby of 3300 g was born to the couple after the treatment with phytoestrogen.

## Conclusions

Long-term administration of phytoestrogens significantly influenced spermatogenesis and improved sperm characteristics, including count and morphology, in a patient with severe oligozoospermia. This enhancement led to the successful completion of an IUI procedure and subsequent achievement of a full-term pregnancy. This favorable outcome suggests a potential therapeutic role for phytoestrogens in managing oligozoospermia. However, further validation through randomized controlled trials is necessary. Addressing severe oligozoospermia symptoms presents challenges, with treatment duration dependent on the underlying etiology. In this case, the patient displayed no identifiable risk factors or concurrent medical conditions contributing to infertility. Lifestyle adjustments and antioxidant supplementation were advised to improve overall sperm health. It is essential to recognize that individual responses to such interventions may vary, requiring ongoing assessment and potential medical interventions.
